# Community Pharmacist Telephonic Medication Reviews with Uncontrolled Asthma Patients: A Pilot Study

**DOI:** 10.3390/pharmacy9010025

**Published:** 2021-01-22

**Authors:** Kathryn A. Hartley, Kendall D. Guthrie, Steven C. Stoner, Justin R. May, D. Matthew Hartwig, Yifei Liu

**Affiliations:** 1 Pharmacy, University of Kansas Health System, Kansas City, KS 66160, USA; khartley@kumc.edu; 2 School of Pharmacy, University of Missouri-Kansas City, Kansas City, MO 64108, USA; stoners@umkc.edu (S.C.S.); liuyif@umkc.edu (Y.L.); 3 Bothwell Regional Health Center, Sedalia, MO 65301, USA; jrmay@brhc.org; 4 Red Cross LTC Pharmacy, Excelsior Springs, MO 64024, USA; mhartwig@rcpharmacy.net

**Keywords:** community pharmacist, asthma, Medicaid, drug-related problems

## Abstract

This study reports the process of telephonic medication reviews conducted by community pharmacists for patients with asthma. The study occurred at an independent community chain in association with a Missouri Medicaid consulting group. Participants were identified utilizing claims data and met the National Quality Forum criteria for uncontrolled moderate-to-severe persistent asthma. A pharmacist performed the initial encounter via telephone which included a knowledge questionnaire, symptom control assessment, and medication review. Pharmacists identified drug-related problems (DRPs) and faxed recommendations to patients’ primary care providers (PCPs). Thirty days later, pharmacists called to follow up with the patients and faxed PCPs to resolve any outstanding DRPs, new DRPs, or recommendations. Questionnaire scores and symptom control assessments were compared and analyzed utilizing a paired t-test, Chi-squared test, or Fisher’s exact test. The number and categories of DRPs, recommendations made by pharmacists, and intervention time were reported. Fourteen participants completed initial encounters with twelve completing follow-up. The majority answered ‘yes’ to at least one symptom control assessment question indicating partially controlled to uncontrolled asthma. The average knowledge assessment score was 5.17 out of 7 initially and 5.42 for the follow-up. Pharmacists identified 43 DRPs and made 41 recommendations with a mean intervention time of 65 min.

## 1. Introduction

Asthma affects nearly 26.5 million Americans and is estimated to cost $56 billion annually ($50.1 billion in direct and $5.9 billion in indirect healthcare costs) [1,2,3]. A patient with asthma may seek emergency care if not properly managed. Approximately 1.7 million emergency room (ER) visits attributed to asthma occur each year [4]. This chronic condition can lead to decreased quality of life, missed days of work for adults, and missed days of school for children. In children 15 years of age and younger, asthma is the third leading cause of hospitalization [5]. In Missouri alone, more than 9.7% of adults and 11.2% of children were diagnosed with asthma in 2014 [6]. Currently, there is no cure for asthma, but it can be controlled with the proper use of inhalers and medications. Unfortunately, the rate of medication non-adherence in asthmatic patients has been reported as 30–70% [7]. 

To improve asthma control and medication adherence, a trusting relationship must be formed between patients and providers. It has been shown that repeated one-on-one contact results in development of trust between patients and healthcare providers [8]. Similarly, frequent interaction in a community pharmacy setting can foster trust between a patient and community pharmacist. The community pharmacist can then educate patients on proper inhaler technique, medication adherence, differences between controller and rescue inhalers, and address any concerns the patient may have such as side effects or financial barriers [9]. Numerous studies have been published examining the impact of pharmacist intervention on asthma outcomes [10,11,12,13,14,15]. In these studies, pharmacists contributed to positive outcomes related to asthma knowledge, adherence, and quality of life [10,11,12,13]. A recent prospective study evaluated the impact of pharmacist-delivered counseling on patients’ knowledge and beliefs about their medications, adherence, and asthma control (n = 90). After three months, there was significant improvement in patients’ knowledge of asthma and medications, attitude towards taking their medications, average asthma control score, and level of adherence [14]. Another study looked at change in Asthma Control Test (ACT) scores before and after a pharmacist intervention (n = 136). Over 13 months, pharmacists provided education, developed asthma action plans, demonstrated inhaler technique, and recommended step up/down therapy. Overall, ACT scores improved from the initial visit until the end of the study [15]. 

Despite the evidence supporting the impact of pharmacists on asthma outcomes, few studies report this impact in the Medicaid population. In 2016, the Centers for Disease Control and Prevention predicted Medicaid or the Children’s Health Insurance Program (CHIP) to have 54,949 visits to the ER out of 145,591 total visits (37.7% of ER visits) [16]. In fact, Medicaid beneficiaries have higher hospital readmission rates and increased utilization of the ER compared to privately paying individuals due to several risk factors and comorbidities. The high utilization of ERs in the Medicaid population is costly to the healthcare system and is significantly more expensive compared with office-based care [17]. Asthma is among the conditions that contribute to ER visits in the Medicaid population. In 2010, Medicaid/CHIP pediatric beneficiaries incurred $272 million in asthma-related ER visits with the average cost per visit at $433. States varied widely in their contributions to this overall cost with Missouri estimated to cost Medicaid between one and ten million dollars for pediatric asthma ER visits [18]. Therefore, there is a need to improve asthma care among MO HealthNet (Missouri Medicaid) patients. 

## 2. Materials and Methods 

### 2.1. Objectives

The study objectives were to (1) report the process of medication regimen reviews conducted by community pharmacists for MO HealthNet patients with uncontrolled moderate-to-severe persistent asthma; (2) describe the number and categories of drug-related problems (DRPs) identified, and the number and categories of recommendations made to providers; and (3) explore the impact of community pharmacist intervention on these asthma patients. 

### 2.2. Setting

At the time of study, Red Cross Pharmacy, Inc. (RCP) was an independently owned community pharmacy chain with fourteen community locations, two long-term care pharmacies, and a durable medical equipment company in northwest and central Missouri. Study participants could be patients of any of the fourteen community pharmacy locations. 

### 2.3. Service Background

The PolyPharmacy Risk Reduction (PPRR) program was developed in June 2016 focusing on the MO HealthNet population. It was a partnership between MO HealthNet, PPRR Program Administrators, and the Missouri Pharmacist Care Network (MO-PCN), a provider network supporting and authorizing pharmacist-provided patient care services in Missouri. The program utilized paid claims data to identify MO HealthNet beneficiaries who were taking twelve or more chronic medications and were therefore at risk for complications and DRPs. Patient cases were assigned to trained pharmacists across the state of MO who reviewed available clinical information and communicated recommendations to the patient’s primary care physician (PCP). With pharmacists’ participation, the program aimed to improve patients’ medication outcomes. Within the original PPRR program, no interaction between the patient and pharmacist was required as the majority of the intervention was completed through provider communication. Participating pharmacists were reimbursed for their services based on service completion time [19]. 

Administrators of the PPRR program wanted to expand the original program to include high-risk patients, such as those with a high rate of readmissions and patients experiencing transitions of care. This pilot study was proposed and approved allowing RCP pharmacists to provide encounters to patients with uncontrolled moderate-to-severe asthma, a population with significant costs to the state. 

### 2.4. Practice Innovation

Prior to conducting the study, four participating pharmacists, one pharmacy technician, and six pharmacy interns received training on study protocols, appropriate utilization of research documents, and current asthma treatment guidelines. PPRR program administrators were responsible for identifying eligible patients utilizing MO HealthNet claims data and sending eligible patients to RCP’s clinical coordinator. The clinical coordinator was a pharmacy technician centrally located at RCP’s corporate office and was highly experienced in the management of pharmacist patient care services. Once eligible patients were transmitted to the clinical coordinator, the coordinator assigned patients to one of the trained pharmacists within the organization. The asthma care process differed from the original PPRR project, because it required direct interaction with patients as well as their providers. 

The service consisted of an initial encounter and a follow-up visit, both completed via telephone. During the initial encounter, the patient verbally consented to the study over the telephone. Through this means of communication, the pharmacist administered an asthma knowledge questionnaire (pre-test), an asthma symptom control assessment from the Global Initiative for Asthma (GINA) guidelines, and performed a comprehensive medication review with specific emphasis on asthma education and inhaler technique [20]. Inhaler technique was described and assessed verbally. The asthma knowledge questionnaire was utilized to gauge a patient’s knowledge of his/her disease state and asthma medications. Investigators developed the questionnaire based on previously published studies and tools [21,22,23,24]. The questionnaire contained seven questions about the participant’s knowledge of his or her disease state, asthma-related medications, and monitoring parameters (Supplementary File S1). The asthma symptom control assessment from the GINA guidelines consisted of four ‘yes’ or ‘no’ questions regarding the patient’s symptom control for the previous four weeks. Depending on the number of ‘yes’ answers, the disease state was categorized as well controlled, partly controlled, or uncontrolled. 

During the comprehensive medication review, the pharmacist identified DRPs and faxed recommendations to the patient’s PCP using a standardized subjective, objective, assessment, and plan (SOAP) note (Supplementary File S2). Thirty days later, the pharmacist followed up with the patient and repeated the asthma knowledge questionnaire (post-test), asthma symptom control assessment, and followed up on recommendations made during the initial encounter. Any unresolved DRPs, new DRPs, or recommendations were faxed to the PCP. Fax was chosen as the preferred provider communication method based on a previous survey study conducted within the same pharmacy organization finding that the majority of family practice physicians registered with MO HealthNet preferred faxed communications for medication therapy management recommendations [25]. The seven DRP categories tracked in this study included unnecessary drug therapy, additional drug therapy needed, ineffective drug therapy, dose too low, dose too high, adverse drug reaction, and non-adherence [26]. The additional drug therapy needed category was further separated to show how many of the DRPs were related to immunizations. Lastly, the RCP clinical coordinator billed the PPRR program directly for each encounter based on service time. 

### 2.5. Study Design

A prospective pilot study was conducted between January 2018 and May 2018. Study subjects were MO HealthNet beneficiaries meeting the National Quality Forum (NQF) criteria for uncontrolled persistent moderate-to-severe asthma. The disease severity was defined as being dispensed at least one prescription for a preferred therapy within the calendar year (anti-asthmatic combinations, antibody inhibitors, inhaled steroid combinations, inhaled corticosteroids, or leukotriene modifiers) and at least one of the following: one emergency department visit with asthma as the principal diagnosis, one inpatient discharge with asthma as the principal diagnosis, four outpatient visits with asthma as a listed diagnosis and two asthma medication dispensing events, or four asthma medication dispensing events. In addition, participants had to be 18 years or older and must have filled at least one medication in the past three months at RCP. Participants excluded from data analysis included dual eligible Medicaid/Medicare beneficiaries; patients with a diagnosis of chronic obstructive pulmonary disease, emphysema, cystic fibrosis, or acute respiratory failure; patients younger than 18 years old; and non-English speaking beneficiaries. 

### 2.6. Evaluation (Impact of Innovation)

The impact was evaluated by reporting the number and type of DRPs identified by a community pharmacist, as well as recommendations provided to PCPs. In addition, the number of recommendations accepted by PCPs was tracked. Asthma knowledge and symptom control between the patients’ initial encounters and follow-ups were assessed by paired t-test, Chi-squared test, or Fisher’s exact test. Pharmacists’ service time was calculated according to the time needed to prepare for the encounter, meet with the patient, and document the encounter. 

## 3. Results

Ninety patients were identified by PPRR program administrators, and out of 90 patients, twenty-four ultimately met inclusion criteria. Primary reasons for not meeting inclusion criteria included falling outside the approved age range (e.g., patients less than 18 years of age) or being deemed dual Medicare-Medicaid eligible after further investigation. Investigators attempted to contact all eligible patients, but eight had non-working telephone numbers and two declined services. Out of twenty-four patients, fourteen consented to the study. All fourteen patients completed the initial encounter, but two were unable to be reached for the follow-up encounter. The majority of patients were female, Caucasian, high school graduates, and the ages ranged from 18–70 years old (Table 1). 

The average initial asthma knowledge assessment score (pre-test) for the twelve patients completing the full study was 5.17 out of 7 points possible, versus an average of 5.42 on the post-test. In regards to the GINA Asthma Symptom Control Assessment, eleven of fourteen patients (nine of the twelve patients completing the full study) answered ‘yes’ to at least one of the four questions at the initial visit, placing them in the partially controlled to uncontrolled categories. For the follow-up visits, four out of twelve patients answered ‘no’ to all symptoms, placing them into the well-controlled category. Using the GINA asthma symptom control test and the asthma knowledge test, pharmacists were able to quickly assess patient’s asthma symptom severity. 

In total, community pharmacists identified 43 DRPs with the majority being needing additional drug therapy (non-immunization and immunization) (Table 2). This averaged to three DRPs per patient. The majority of non-immunization DRPs were unnecessary drug therapy. Pharmacists sent 41 recommendations to the patients’ PCPs. The majority of recommendations made were to recommend and/or provide immunizations to patients. Additionally, recommendations were made to start five patients on a controller inhaler to help better improve their asthma symptoms. Out of those five recommendations, one of the recommendations was accepted by the patient’s primary care provider, and the patient was prescribed and picked up a controller inhaler. For patients experiencing adherence issues with their inhalers, recommendations were made to assess and reinforce adherence to controller inhalers at follow-up appointments. Lastly, pharmacists recommended discontinuing excessive or unnecessary drug therapies. Six recommendations were accepted by providers faxing forms back to the pharmacy. Pharmacists were unable to assess if the immunization recommendations were accepted. On average, it took pharmacists 65 min to complete the entire patient encounter including workup and documentation (Table 1). 

## 4. Discussion

As mentioned previously, few studies have looked at the impact of community pharmacists on asthma patients within the Medicaid population. In this study, on average, community pharmacists identified three DRPs per patient. Similarly, in a study conducted by Haugbolle et al., an average of four DRPs per patient were identified in patients with angina pectoris, type 2 diabetes, and asthma, with the most common problem being inappropriate use of medications [27]. In contrast to our study, Haugbolle et al. conducted the patient interviews at the patient’s home where they were able to observe inhaler technique, read body language, interpret level of education, and observe wheezing or shortness of breath [27]. These factors were more difficult to assess over the phone as it was up to the discretion of the patient to explain their technique, symptoms, and condition. 

In our study, the majority of patients demonstrated the need for education and/or intervention, showcasing an opportunity for pharmacist service development. For example, seventy-nine percent of patients answered ‘yes’ to at least one question on the GINA Asthma Symptom Control Assessment indicating they were uncontrolled, and sixty-four percent missed at least one question on the asthma knowledge questionnaire. This allowed community pharmacists the opportunity to provide targeted education to each patient. While we did see a small increase in the number of patients with controlled asthma based on the symptom control assessment, statistical significance was not found likely due to the small sample size. However, in a larger Australian study with community pharmacists involved in an asthma care program, pharmacist intervention and education significantly improved overall asthma control. Patients who were educated by a pharmacist were 2.7 times more likely to improve from ‘severe’ to ‘not severe’ than the control group [11]. Results also showed community pharmacists helped improve overall adherence, asthma knowledge, perceived control of asthma questionnaires, an increased usage of the controller inhaler, and a decreased usage of the rescue inhaler [11]. 

The highest number of drug-related problems and recommendations in our study population pertained to patients needing additional immunizations. There are several immunizations that are very important in patients with an asthma diagnosis, including pneumococcal and influenza vaccinations. These results were not surprising given the suboptimal vaccination rates in the asthma population reported by the CDC. In 2015, the CDC stated that 46.7% of adults with asthma admitted to receiving the influenza vaccine within the past year. These results varied by state with 51.8% of Missouri residents with asthma reporting receiving the vaccination [28]. In a study conducted by Klassing et al., pharmacists identified many adult patients with asthma and chronic obstructive pulmonary disease who needed influenza and pneumococcal vaccinations further supporting the impact of pharmacists in these patient populations [29]. The results from our study further emphasize the impact pharmacists can have on identifying patients at risk for vaccine preventable diseases and the important role pharmacists play in screening and administering immunizations. 

Through the two interventions speaking with the patients via telephone, pharmacists were provided the opportunity to identify and interact with patients experiencing uncontrolled asthma. Patients were educated about the importance of adherence, how to use inhalers, where they fell on the asthma symptom control, and the need to discuss their symptoms with their prescribers. Chronic conditions and medications can often be overwhelming for patients, and service designs similar to this allowing patients dedicated time to focus on their condition and receive tailored education may help patients feel more in control of their health. Improved asthma control could lead to an improved medication experience for these patients, and further research with larger sample sizes is critical. 

In our study, although it took an average of 65 min for pharmacists to complete the entire patient encounter, pharmacists were able to perform a thorough assessment and clinically evaluate each patient’s asthma symptoms as well as other drug-related problems. After a relationship with a patient is established, the time for each encounter should decrease. Of note, this service was primarily completed by the site’s community pharmacy resident as part of the residency learning experience so service time may differ when incorporating full-time, experienced pharmacists. 

## 5. Limitations

There were several limitations. First, the sample size was limited. Many patients were initially identified but subsequently removed due to meeting exclusion criteria. Out of the 90 patients identified by PPRR program administrators, 56 were excluded based solely on age. In addition, it was difficult to reach patients by telephone due to non-working numbers or unanswered voicemails. Second, no standard counseling instructions were given to pharmacists when educating patients on the items they missed on the questionnaires. Patients may have not received focused education regarding the missed item, leading to them missing the same item on the post-test. Third, for each unanswered recommendation, a phone call was made to the provider’s office. However, most attempts were unsuccessful. Therefore, the amount of accepted recommendations was limited. In a community pharmacy setting without access to electronic medical records, communication was restricted to telephone and facsimile which is often inefficient. Furthermore, it was difficult to define the clinical significance of the identified DRPs until prescriber acceptance rates were determined. Future research should also look at the impact of telephonic service designs on patient-provider trust development and patient acceptance of recommendations. While the telephonic service design presented some challenges, such as assessing inhaler technique, telehealth service designs are becoming increasingly utilized and delivered making the investigation of these contact methods vital.

## 6. Conclusions

Pharmacists were able to educate uncontrolled asthma patients about their disease state and asthma medications. They made recommendations to physicians to optimize medication regimens for patients. Furthermore, the largest number of drug-related problems and recommendations pertained to patients needing additional vaccinations, emphasizing the important role of pharmacists in the vaccination screening and administration process. This service design, with necessary modifications, could serve as an example to extend to different state Medicaid programs as well as help implement programs for other chronic disease states. For the future, modifications could include expanding inclusion criteria (such as including pediatric patients) to incorporate more participants into the service, collaborating directly with individual PCP offices to enhance bidirectional communication, and setting up follow-up appointment times with patients at the time of the initial encounter to prevent patients being lost to follow-up. Furthermore, it may have been helpful for patients to receive communication directly from the Medicaid program to aid with subject recruitment.

## Figures and Tables

**Table 1:** Participant demographic information and intervention time.

Participant Demographic Information (n = 14)	
Age in Years	
Mean (Range)	38.7 (18–70)
Median	35.5
Gender [n (%)]	
Male	1 (7.1%)
Female	13 (92.9%)
Race [n (%)]	
Caucasian	10 (71.5%)
African American	2 (14.3%)
Other	1 (7.1%)
Unknown	1 (7.1%)
Educational Level [n (%)]	
Some High School	3 (21.4%)
High School Diploma	4 (28.6%)
Some College	7 (50%)
Number of Medications (OTCs, herbals, and prescriptions)	
Mean (Range)	11.1 (0–35)
Median	10
Pharmacist Time (in minutes)	
Preparation:	
Mean (Range)	22.9 (10–60)
Encounter:	
Mean (Range)	24.3 (15–45)
Documentation:	
Mean (Range)	17.9 (10–30)

OTC: over-the-counter.

**Table 2:** Drug-related problems identified.

Drug Related Problems (n = 43)	
Additional Drug Needed Immunization	18
Additional Drug Needed Non-Immunization	6
Unnecessary Drug Therapy	8
Inability to be Adherent	7
Ineffective or Suboptimal Drug	4
Total	43

## Data Availability

All relevant data is contained within the article. No other data is publicly available to maintain patient privacy and confidentiality.

## Supplementary Materials

The following are available online at https://www.mdpi.com/2226-4787/9/1/25/s1, File S1: Asthma Questionnaire, File S2: Standard Asthma Documentation Form.
