# Supplementary material for: Community Pharmacist Telephonic Medication Reviews with Uncontrolled Asthma Patients: A Pilot Study
Source: Pharmacy (Basel). 2021 Jan 22;9(1):25. doi: 10.3390/pharmacy9010025 (PMC7838915; doi:10.3390/pharmacy9010025)
Supplement: Supplementary file 1 [file pharmacy-09-00025-s001.zip › pharmacy-1038477-supplementary/Supplementary Files/Supplementary File S1.docx]

**Asthma Questionnaire**

1. Which of the following causes asthma?
2. Narrowing of airways
3. Too large of lungs
4. Too small of lungs
5. All of the above
6. How often should you use your rescue inhaler?
7. Twice daily
8. Everyday
9. As needed for wheezing/shortness of breath
10. Four times a month
11. Which of the following can trigger asthma?
12. Exercise
13. Allergens (pollen, mold, dust, pet dander)
14. Strong Odors (perfumes, tobacco smoke, etc)
15. All of the above
16. How often should you use your controller inhaler?
17. Only during allergy season
18. Everyday
19. As needed for wheezing/shortness of breath
20. Four times a month
21. Which of the following is not a sign of asthma?
22. Wheezing
23. Tightness in the chest
24. Shortness of breath
25. Stomach pain
26. Which of the following can be used at home to monitor breathing and detect worsening asthma?
    1. Chest X-Ray
    2. Peak flow meter
    3. Blood pressure cuff
    4. Ultra sound of your lungs
27. Asthma action plans are only for individuals who have frequent shortness of breath.
    1. True
    2. False
