# Supplementary material for: Community Pharmacist Telephonic Medication Reviews with Uncontrolled Asthma Patients: A Pilot Study
Source: Pharmacy (Basel). 2021 Jan 22;9(1):25. doi: 10.3390/pharmacy9010025 (PMC7838915; doi:10.3390/pharmacy9010025)
Supplement: Supplementary file 1 [file pharmacy-09-00025-s001.zip › pharmacy-1038477-supplementary/Supplementary Files/Supplementary File S2.docx]

**Standard Asthma Documentation Form**

**Demographics:**

| Patient’s name: | |  | | | | | | | | | | | | | |
| --- | --- | --- | --- | --- | --- | --- | --- | --- | --- | --- | --- | --- | --- | --- | --- |
| DOB: |  | | | | Phone Number: | |  | | | | | Gender: Male Female | | | |
| PCP: |  | | | | Phone: |  | | | | | Fax: | |  | | |
| Race/Ethnicity: | |  | | | Highest Education Level: | | |  | | | | | | | |
| Time Spent: | | | Preparation: |  | | | Encounter: | |  | | | | | Documentation: |  |
| Contact Attempts: | | | 1^st^ Attempt: |  | | | 2^nd^ Attempt: | | |  | | | | 3^rd^ Attempt: |  |

**Subjective:**

HPI:

PMH:

Home Medications: see attached

**Objective:** An asthma educational test was done with the patient to gauge her baseline knowledge.

|  | **Educational Test Score** |
| --- | --- |
| **pre** |  |
| **post** |  |

**Assessment/Plan:**

**Recommendations to the Prescriber:**

|  |
| --- |
|  |
|  |
| \| **Pharmacist Signature:** \|  \| \| --- \| --- \| |

**Prescriber Response/Comments:**

|  | | |
| --- | --- | --- |
| **Physician Signature:** |  |  |
